# An asymmetric protoplast fusion and screening method for generating celeriac cybrids

**DOI:** 10.1038/s41598-021-83970-y

**Published:** 2021-02-25

**Authors:** Silvia Bruznican, Tom Eeckhaut, Johan Van Huylenbroeck, Ellen De Keyser, Hervé De Clercq, Danny Geelen

**Affiliations:** 1 Flanders Research Institute for Agriculture, Fisheries and Food (ILVO) – Plant Sciences Unit, Melle, Belgium; 2 Department Plants and Crops, Ghent University, Ghent, Belgium

**Keywords:** Plant breeding, Agricultural genetics

## Abstract

Celeriac F_1_ hybrid seed production is currently complicated due to the instability of cytoplasmic male sterile lines. To develop alternative alloplasmic CMS lines, an asymmetric protoplast fusion and hybrid screening methodology was established. Celeriac suspension cells protoplasts were used as the acceptor and carrot, coriander and white celery mesophyll protoplasts as the donor for protoplast fusion experiments. Acceptor cytoplasmic inheritance was inhibited by iodoacetamide treatment and donor nuclear genome inheritance was prevented by UV exposure. Protoplasts were selectively stained and fused using electroporation and polyethylene glycol, and candidate hybrid shoots were obtained. One chloroplast and three mitochondrial markers that could distinguish acceptor and donors organelles were used to characterize over 600 plants obtained after fusion events, without identifying any cybrid. In order to increase the testing efficiency a high number of micro plantlets were pooled and hence the presence of the carrot specific *Atp1* marker in one of the pooled samples was detected. We demonstrated that fusion took place between celeriac and a carrot indicating that the creation of viable hybrids is possible although at a very low frequency. These findings open the path for new cytoplasmic hybridization and the isolation of novel CMS lines of celeriac.

## Introduction

*Apium graveolens* var. *dulce* (celery) and var. *rapaceum* (celeriac) markets are dominated by open pollinated cultivars, that are often characterized by lack of uniformity, which can be tackled by developing hybrid seed. A current bottleneck in celery and celeriac hybrid breeding is the poor quality of the available male sterile lines. Genetically encoded male sterility (GMS) and cytoplasmic male sterility (CMS) are used to bypass the tedious work of umbel flower emasculation. CMS is the preferred material because it is maternally inherited allowing better control of hybrid seed production. Two independently isolated male sterile (MS) lines are available for *Apium*, one is a wild accession from Iran, ‘P1229526’^1^, and another one was discovered in a commercial field of the inbred line ‘01–3’^2^. CMS was introgressed in celery from a wild relative as Dawson reported in 1993 to the ‘Grower’ magazine, but is unstable. However, none of these lines are currently used for mass scale seed production. Despite the lack of an efficient CMS system, the number of hybrid cultivars available on the market increases, corroborating the need for uniformity in growth. The absence of a stable CMS line within the public domain has prompted us to develop protoplast hybridization methodology for *Apium* species.

By combining the cytoplasm of a donor of one species with the nucleus of an acceptor belonging to another species, interspecies nuclear and organelle genomic configurations are created. These so-called cybrids occasionally display CMS as a result of the incongruence of nuclear and cytoplasmic DNA expression. The potential of asymmetric protoplast fusion to create CMS was demonstrated in a number of species. Forsberg, et al.^3^ produced alloplasmic CMS *Brassica napus* by fusion of *B. napus* intact protoplasts with UV irradiated *Arabidopsis thaliana* protoplasts, which were later characterized by Teixeira, et al.^4^. Iodoacetamide (IOA) inactivated *B. napus* protoplasts fused with X-ray treated *B. tournefortii* resulted in alloplasmic *B.napus* CMS that was caused by mitochondrial gene rearrangements^5^. Alloplasmic CMS has been obtained in *Nicotiana* after fusing intact *N. sylvestris* protoplasts with X-ray treated *N. rustica* protoplasts^6^, and intact *N. tabacum* protoplasts with X-ray irradiated *N. africana* protoplasts^7^. Melchers, et al.^8^ generated novel CMS types in tomato by fusing *Lycopersicon esculentum* IOA treated protoplasts with *Solanum acaule* or *Solanum tuberosum* **γ** or X-ray irradiated protoplasts. They obtained fertile plants only when the donor was *Solanum lycopersicoides*, in spite of the presence of donor mitochondria fragments in the acceptor. The donor genotype is thus important and, in this case, determined the potential to create a new CMS type. Asymmetric protoplast fusion is routinely used in the *Citrus* genus, a model for somatic hybridization, where attempts were made to transfer CMS from Satsuma mandarin to other seedy cultivars^9^. Moreover, cybrids with different mitochondrial and chloroplast combinations were recovered in citrus following symmetric fusions between Chios and Clementine and Chios and Sanguinelli^10^. The success of an asymmetric protoplasts fusion and subsequent regeneration depends on the integration of a series of preparatory steps that each require optimization. In general, a protoplast regeneration protocol for the acceptor species is preferably available or needs to be developed prior to setting up fusion experiments. Previously, we reported a protocol for the regeneration of celery protoplasts derived from embryogenic cell suspension var. *dulce* and *rapaceum*^11^. A second pre-requirement is the availability of a method to inactivate the nuclear DNA from the donor and the cytoplasmic DNA from the acceptor. Finally, a method for protoplast fusion is needed to create the interspecies hybrids. In celery, a few reports describe protoplasts fusion, and both electrofusion and chemical (PEG mediated) fusion were employed^12,13^. Wang, et al.^14^ reported the regeneration of 3 hybrids after a symmetric fusion between celery mesophyll and *Daucus carota* (carrot) root protoplasts. Tan, et al.^13^ performed asymmetric fusion between IOA treated celery and UV treated carrot protoplasts and regenerated 11 petaloid celery CMS plants.

Cytoplasmic genome characterization of the plants regenerated from fusion events is especially important when asymmetric fusions are employed with the purpose of chloroplast or mitochondria integration into the acceptor. Molecular markers from both nuclear and cytoplasmic genomes are widely used for confirming the hybridity of shoots regenerated from fusions. It is a rapid and cost-effective method that identifies regions in the genome of the hybrid that are polymorphic between the donor and the acceptor species. An alternative approach consists of high resolution melting analysis (HRM), a fast method for plasmotype discrimination based on the presence of SNPs, insertions and deletions (INDELS) or SSRs^15^.

Here, we report on the development of asymmetric protoplast fusion and regeneration to establish alloplasmic CMS types in celeriac. Asymmetric fusion and regeneration were optimized and resulting cybrids characterized by means of mitochondrial and chloroplast genetic markers. The efficiency of different methods of selective protoplast inactivation, fusion, regeneration and hybridization are reported and discussed.

## Results

### Experiment 1: UV inactivation of donor protoplasts

The viability immediately after isolation of ‘Parmex’ carrot*, Coriandrum sativum* (coriander) and ‘WL253’ white celery protoplasts was 63, 54, and 48% (Table 1). Upon 7 days of cultivation, the protoplast viability declined to 38, 30 and 13% respectively. The prevent transmission of the nuclear DNA of the donor protoplasts, freshly isolated protoplasts were treated with UV for 2, 4, and 6 min. The UV-induced damage was tolerated quite well initially but led to loss in viability after 7 days (Table 1). A 6 min UV treatment reduced ‘Parmex’ carrot protoplast viability to 10.7% and coriander protoplasts to 8.1%. The reduction in viability was statistically significant compared to untreated cells for ‘Parmex’ carrot and coriander but not for ‘WL253’ white celery.

**Table 1:** Viability (%) of donor (carrot, coriander and white celery) mesophyll protoplasts upon UV treatment.

Donor	UV treatment	Viable protoplasts ± SE (%)	
		After isolation	After 7 days
‘Parmex’ carrot	Untreated	63.3 ± 1.1 a	38.7 ± 1.8 a
	2 min	62.1 ± 1.5 a	17.9 ± 1.5 b
	4 min	59.1 ± 1.6 a	16.8 ± 1.3 b
	6 min	59.6 ± 1.2 a	10.7 ± 1.3 c
Coriander	Untreated	54.9 ± 1.6 a*	30.2 ± 2.0 a
	2 min	52.3 ± 2.3 a*	15.9 ± 1.8 ab
	4 min	50.9 ± 1.6 a*	13.5 ± 1.6 b
	6 min	52.0 ± 2.3 a*	8.1 ± 0.9 c
‘WL253’ white celery	Untreated	48.8 ± 1.4 a	13.2 ± 3.2 a*
	2 min	45.0 ± 1.7 a	6.4 ± 0.9 a*
	4 min	44.9 ± 2.0 a	7.0 ± 1.2 a*
	6 min	45.5 ± 2.0 a	5.8 ± 1.1 a*

Data shown are means (n = 6, ± SE).

a, b, c significant differences based on Tukey’s honest significant test, p < 0.05.

a* no significant differences between the groups based on Welch’s robust tests of equality of means, p < 0.5.

Since 6 min of exposure to UV did not cause a significant viability loss immediately after the treatment, this condition was considered as a suitable pre-treatment for inactivating the nuclear DNA of the donor protoplasts (see experiment 5).

### Experiment 2: IOA inactivation of acceptor protoplasts

To prepare acceptor protoplasts from the ‘Diamant’ celeriac variety, we initiated cell suspension cultures from callus and maintained 4 lines : 23, 24, 25 and 26. The viability of freshly isolated protoplasts assessed by FDA staining of these 4 lines was 77, 83, 86, and 83% respectively and 25, 56, 43 and 40% respectively after 7 days (Table 2). To asses mitochondria inactivation, protoplasts were treated with IOA for 10, 15 and 20 min. Under these conditions, viability significantly reduced for the lines 24, 25 and 26 while line 23 tolerated IOA treatment. Further incubation for 7 days revealed that a 20 min IOA treatment was lethal for line 23, 25 and 26. Cell line 24 was quite tolerant compared to the other cell lines with 33% of the protoplasts alive after IOA treatment (Table 2). A 10 min IOA treatment was not lethal to any of the cell lines (Table 2). Taken together, it follows that the duration of IOA treatment needed to be adjusted depending on the cell line which was used in subsequent experiments 4 and 5.

**Table 2:** The viability frequencies (%) of acceptor (celeriac ‘Diamant’) suspension protoplasts upon IOA treatment.

Cell line	IOA treatment	Viability frequency ± SE (%)	
		After isolation	After 7 days
Celeriac ‘Diamant’ 23	Untreated	77.0 ± 2.8 a	25.2 ± 3.0 a
	10 min	75.1 ± 4.1 a	16.3 ± 3.4 a
	15 min	79.5 ± 6.5 a	0.0 ± 0.0 b
	20 min	75.5 ± 3.1 a	0.0 ± 0.0 b
Celeriac ‘Diamant’24	Untreated	83.8 ± 0.2 a	56.3 ± 1.5 a
	10 min	78.0 ± 1.0 ab	55.5 ± 1.2 a
	15 min	79.0 ± 2.7 ab	52.8 ± 1.9 a
	20 min	75.0 ± 1.1 b	33.1 ± 2.3 b
Celeriac ‘Diamant’25	Untreated	86.2 ± 3.7 a	43.8 ± 0.9 a
	10 min	72.9 ± 0.9 ab	5.7 ± 0.5 b
	15 min	67.3 ± 4.16 b	0.0 ± 0.0 c
	20 min	62.7 ± 5.3 b	0.0 ± 0.0 c
Celeriac ‘Diamant’ 26	Untreated	83.5 ± 3.0 ab	40.7 ± 6.1 a
	10 min	86.6 ± 0.6 a	29.8 ± 3.1 ab
	15 min	75.3 ± 4.7 ab	21.2 ± 4.8 b
	20 min	74.1 ± 1.6 b	0.1 ± 0.1 c

Data shown are means (n = 6, ± SE).

a, b, c significant differences based on Tukey’s honest significant test, p < 0.05.

### Experiment 3: Fluorescent markers to monitor fusion events

**Figure 1:**
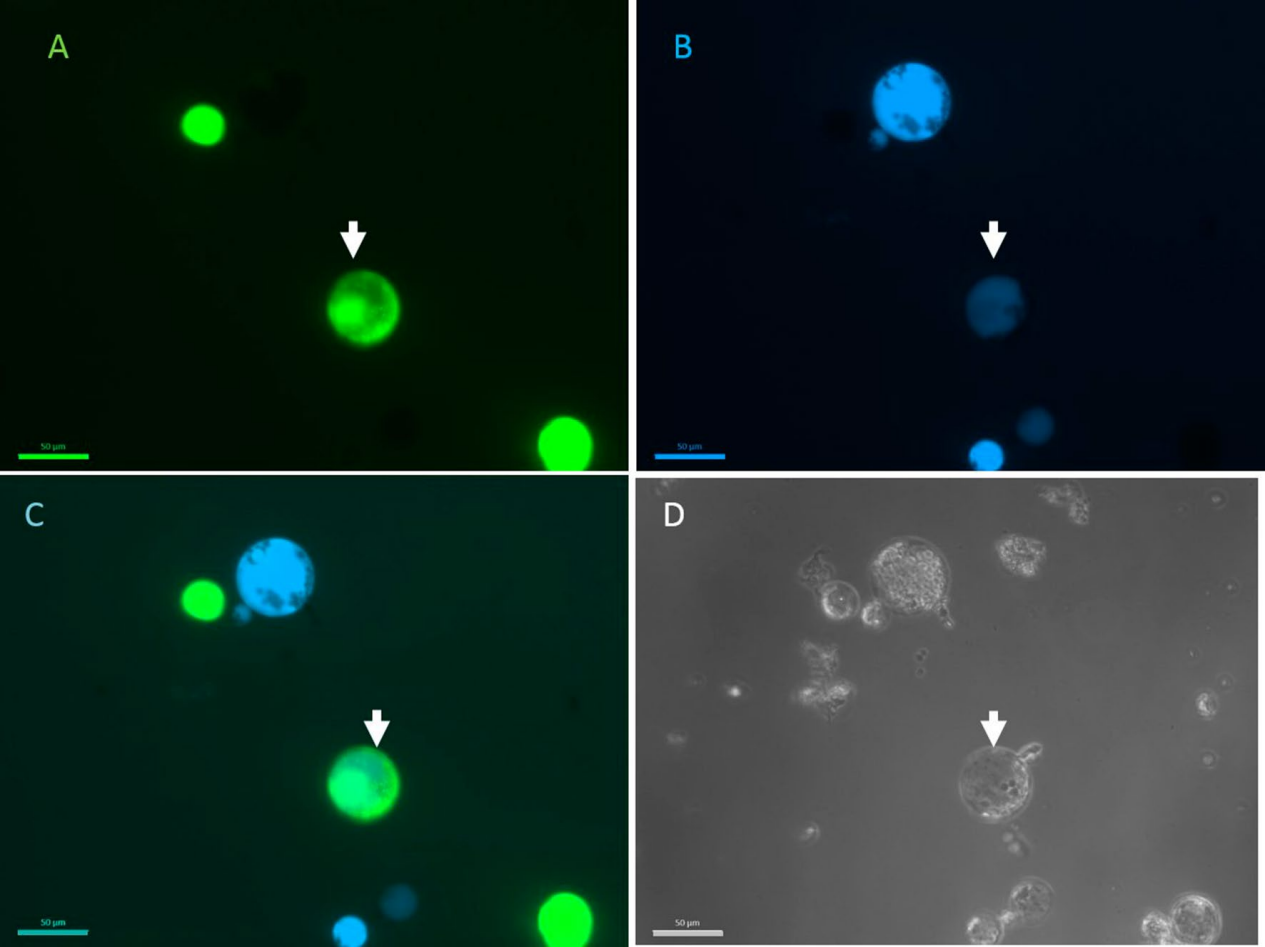
Celeriac and coriander protoplast fusion. Celeriac protoplasts were stained with FDA (**A**) and coriander protoplasts with calcein blue (**B**). Overlap between the photos taken with separate filters showing dual staining (**C**). All cells were visible in bright field (**D**). The arrow indicates the hybrid cell. The scale bar is 50 µm.

To verify whether the fusion techniques used are able to induce fusions between the acceptor and the donor genera, we prepared differently stained protoplasts. Donor protoplasts were stained with FDA and acceptor protoplasts with calcein blue (Fig. 1). To test whether fused protoplasts show dual staining, fusion reactions were performed between ‘Diamant’ 24 celeriac suspension protoplasts and ‘Parmex’ carrot, coriander and ’Wl253’ white celery mesophyll protoplasts. Cells stained with calcein blue and FDA were observed after the electroshock and chemical fusion in all donor:acceptor combinations, indicating that fused cells contain both fluorescent dyes (Fig. 1).

### Experiment 4: preliminary protoplast fusions

We performed a series of preliminary electrofusions of acceptor protoplasts from celeriac ‘Diamant’ (cell lines 23, 24 and 25), and from celeriac ‘Daybreak’ 4.1 with coriander and carrot ‘Dolanka’, ‘Parmex’ and ‘Karotan’. The different times of IOA and UV treatments are presented in Table 3. The electrofusion conditions were also varied, applying different fusion alignments and electric pulse voltages (Table 3). Within 16 weeks, shoots were obtained in 3 out of 12 tests (D, F and G) (Table 3). The fusion alignment voltage and the number of fusion pulses did not influence the capacity to induce callus. Because a 25 min IOA exposure led to successful shoot regeneration this duration was used in the subsequent experiments (experiment 5).

**Table 3:** Overview of parameters applied in the electrofusion of experiment 4.

Fusion	Acceptor		Donor		Acceptor : donor ratio	Electrofusion		Regeneration	
	Cell line	IOAz	Cultivar/accession	UVCz		ACy	DCx	Acceptor	Fusion
A	Celeriac ‘Diamant' 23	5	Carrot ‘Dolanka'	3	1: 1	3 V; 40 s	25 V; 60 µs; 1	Died	Callus
B	Celeriac ‘Diamant' 23	10	Carrot ‘Dolanka'	3	1: 1	3 V; 40 s	25 V; 60 µs; 1	Died	Died
C	Celeriac ‘Diamant' 23	20	Carrot ‘Dolanka’	3	1 : 1	3 V; 40 s	25 V; 60 µs; 1	Died	Died
D	Celeriac ‘Diamant' 24	20	Carrot ‘Dolanka'	6	1 : 1	3 V; 40 s	25 V; 60 µs; 1	Shoots	Shoots
E	Celeriac ‘Diamant' 24	20	Carrot ‘Dolanka'	6	1 : 2	3 V; 40 s	25 V; 60 µs; 1	Died	Died
F	Celeriac ‘Diamant' 24	25	Carrot ‘Parmex'	6	1 : 1	2.5 V; 40 s	25 V; 60 µs; 2	Shoots	Shoots
G	Celeriac ‘Diamant' 24	25	Carrot ‘Parmex'	6	1: 2	2.5 V; 40 s	25 V; 60 µs; 2	Shoots	Shoots
H	Celeriac ‘Diamant' 24	25	Coriander, csR1	6	1 : 1	3 V; 40 s	25 V; 60 µs; 2	Died	Died
I	Celeriac ‘Diamant' 24	30	Carrot ‘Parmex'	6	1 : 2	3 V; 40 s	25 V; 60 µs; 1	Died	Died
J	Celeriac ‘Diamant' 25	20	Carrot ‘Karotan’	6	1 : 2	3 V; 40 s	25 V; 60 µs; 1	Died	Died
K	Celery ‘Daybreak' 4.1	20	Carrot ‘Dolanka'	2	1 : 1	3 V; 40 s	25 V; 60 µs; 2	Died	Died
L	Celery ‘Daybreak' 4.1	20	Carrot ‘Dolanka'	2	1 : 1	3 V; 40 s	50 V; 60 µs; 2	Died	Died

Per electrofusion the regeneration status of the IOA control and the fusion samples, 16 weeks after the start of the culture, is shown.

^z^Exposure time (min).

^y^Alternating current: alignment voltage; alignment duration.

^x^Direct current: pulse voltage; pulse duration; number of pulses.

Regeneration of shoots was obtained in fusions that involved ‘Diamant’ cell line 24 as the acceptor, and carrot ‘Dolanka’ and ‘Parmex’ as donors. Hence, the acceptor material was more important than the donor material. ‘Diamant’ cell line 24 was selected as the acceptor partner in the following fusions (experiment 5).

### Experiment 5: subsequent protoplast fusions

Based on previous experiments, a series of electrofusions and chemical fusions between ‘Parmex’ carrot, coriander and ‘WL253’ white celery donor protoplasts and celeriac (‘Diamant’ cell line 24) acceptor protoplasts were performed using 6 min UV and 25 min IOA treatment (Table 4). There was no significant difference in the cell recovery percentage between the fusion methods employed (41.6% for electrofusion and 51.8% for PEG) or between the different donor species (42% for carrot, 50.5% for coriander and 47.6% for white celery) fused with celeriac suspension (data not shown). The protoplasts of the UV-treated and untreated donors died within 1 to 3 weeks after culture in all fusion conditions tested. The untreated celeriac cell suspension derived protoplasts started to divide during the first week of culture and developed into microcolonies and callus, producing the first shoots around 9–10 weeks after the start of the cultures throughout the experiments.

**Table 4:** Asymmetric electrofusion and PEG fusion between ‘Diamant’ celeriac (acceptor) and ‘Parmex’ carrot, coriander or ‘WL253’ white celery (donors) protoplasts performed in experiment 5.

Fusion type	Biological replicate	Regeneration of IOA treated celeriac	# Shoot-producing technical replicates / # total technical replicates		
			Celeriac: carrot	Celeriac: coriander	Celeriac: white celery
PEG fusion	1	Shoots	1/2	4/4	3*/4
	2	Shoots	2*/3	3/3	4/4
	3	Shoots	2*/3	3/3	2/4
	4	Shoots	2/3	3/3	3/3
Electrofusion	1	Callus	0/3	1/3	1/3
	2	Shoots	2/3	3/3	3/3
	3	Single cells	0/4	0/4	0/4
	4	Microcolonies	0/3	2/3	2/3

The number of shoot-producing technical replicates / number of total technical replicates, per fusion type and independent repeat are depicted. The data are recorded 12 weeks after the start of the culture.

*One replicate contaminated before it was able to produce shoots.

In the PEG tests, the IOA treated celeriac protoplasts started to divide at the same time as the untreated celeriac protoplasts, although less colonies were formed, and induced shoots after 9–11 weeks. Shoots were regenerated from all 4 chemical fusions performed between celeriac and every donor (Table 4). The celeriac : white celery chemical fusion 3 produced less callus and initiated shoots in only half of the replicates. Since the initial cell number after fusion was overestimated, the cell density of the culture was too low and disabled culture development at a fast rate.

The regeneration of IOA celeriac protoplasts varied strongly between the 4 electrofusion tests and shoots were obtained in only one of the tests, electrofusion 2 (Table 4). This was reflected in a lower number of fusion-derived repetitions that produced shoots (Table 4). However, sufficient shoots were recovered from all acceptor:donor electrofusion combinations.

### Molecular characterization

**Figure 2:**
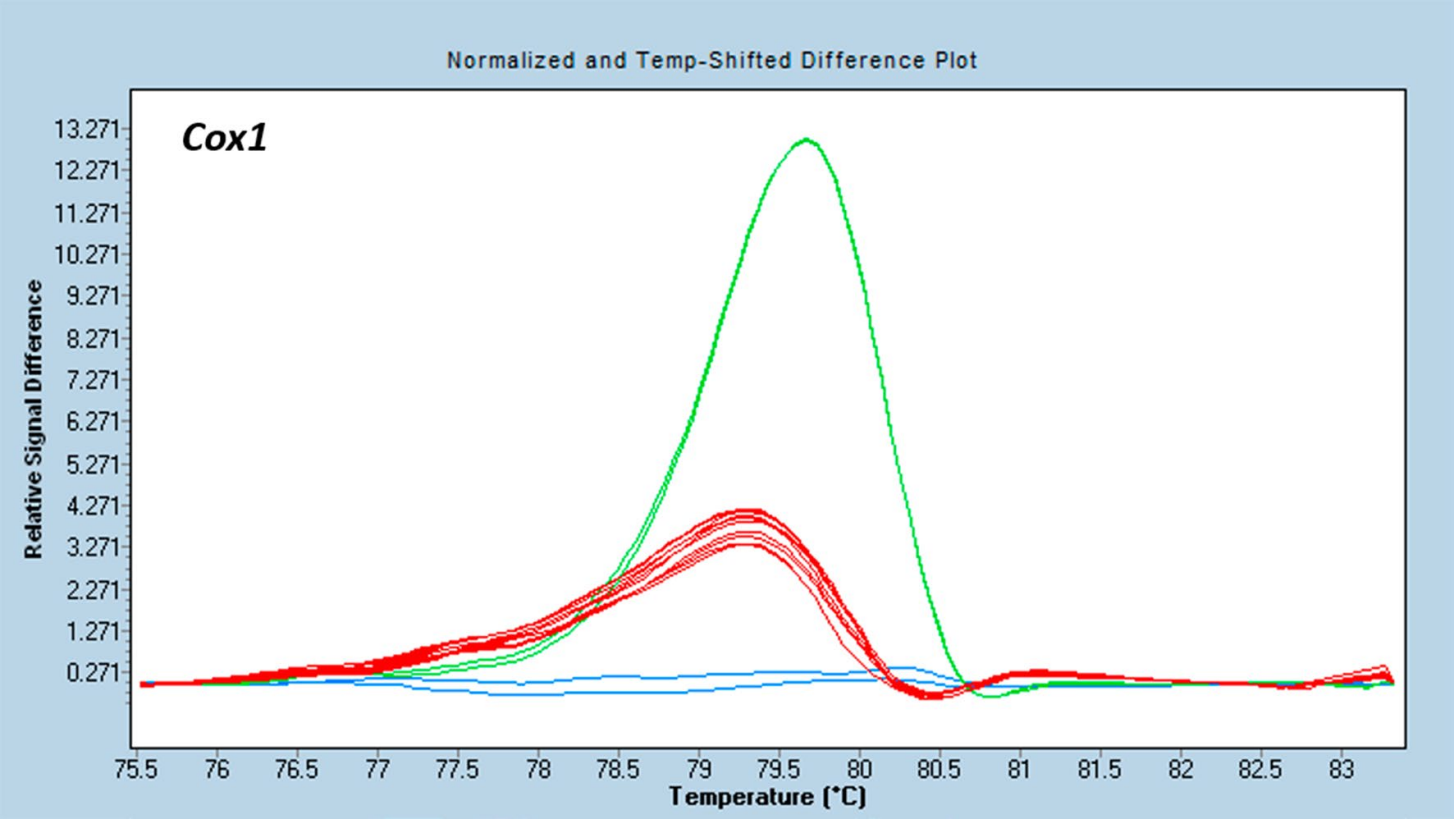
Discrimination between the plasmotypes of the different genera used during fusion experiments with the HRM marker *Cox1*. Distinct coloured curves are significantly different as calculated by the Gene Scanning software (Roche Diagnostics). Melting profiles differed between carrot ‘Dolanka’ (green), carrot ‘Parmex’ (red) and celeriac ‘Diamant’ (blue).

In the HRM analysis, the *Cox1* region amplified in all used donors and in the celeriac acceptor. The differences in the melting profiles allowed to distinguish the mitochondria of celeriac ‘Diamant’ and carrot ‘Dolanka’ and ‘Parmex’ (Fig. 2). The melting profiles of celeriac on the one hand and coriander or ‘WL253’ white celery on the other hand were not different (data not shown).

The regions *Atp1* and *Atp9* only amplified in carrot and coriander, but not in white celery and the acceptor celeriac ‘Diamant’ (Supplementary Table 1). As such both *Atp1* and *Atp9* serve as an ‘on–off’ marker system to distinguish between carrot and coriander donors and acceptor. However, ‘Diamant’ celeriac and ‘WL253’ white celery plasmotypes were not distinguished by these markers.

An overview of the putative somatic hybrid samples, regenerated from Experiment 4 and 5, and the combination of markers used is given in Table 5.

**Table 5:** Overview of the fusion derived samples, originating from Experiment 4 and 5, analysed with mitochondrial (*Cox1, Atp1* and *Atp9*) and chloroplast (DcMP) markers.

Donor	Fusion type	# Samples (5 plantlets pooled)	# Samples (microplants pooled per jar)	Markers tested			
				*Atp1*	*Atp9*	*Cox1*	*DcMP*
‘Parmex' carrot	Electrofusion	50	8	√	√	√	√
	PEG	20	9	√	√	√	√
‘Dolanka' carrot	Electrofusion	11	6	√	√	√	√
Coriander	Electrofusion	20	4	√	√	*x*	√
	PEG	22	8	√	√	*x*	√

The acceptor line was celeriac ‘Diamant’ in all cases. The number of analysed plants and the markers checked are indicated.

**Figure 3:**
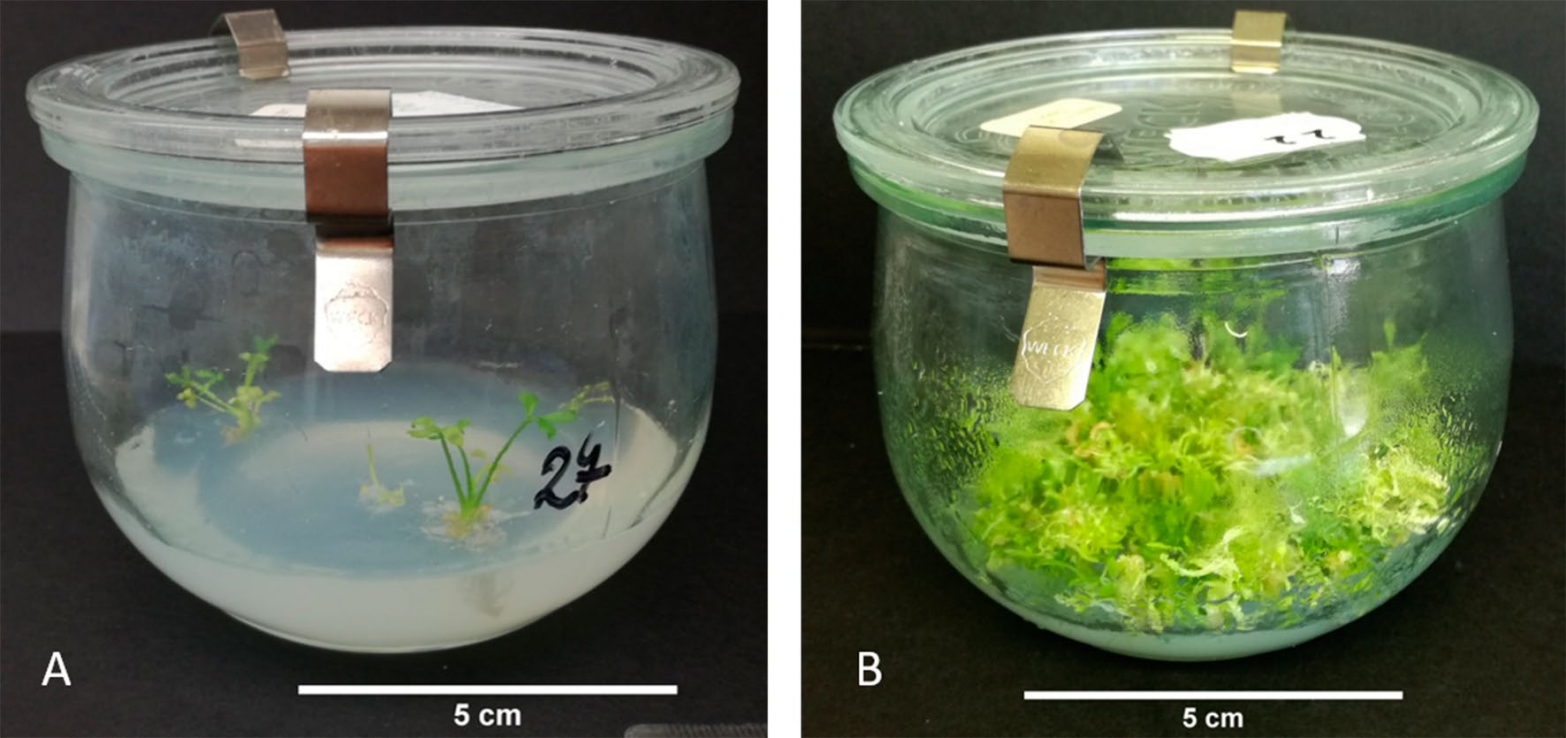
Type of samples used for molecular characterization. (**A**) Rooting plants were pooled by 5 per sample; (**B**) very tiny plants that were not rooting and were growing in bundles were pooled per jar, (microplant samples). The scale bar is 5 cm.

None of the fusion derived samples tested had a melting profile similar to that of the donor carrot, neither an intermediate profile that would suggest a combination of both types was found for *Cox1. F*or the *Atp1* region, a ‘Dolanka’ carrot electrofusion D (Table 3) derived sample (sample 22, Supplementary Table 1), amplified. The amplification of this sample appeared later (Cq 31) than carrot (Cq 16) but clearly differed from the NTC (Cq 37). For the *Atp9* region, a coriander (sample 131, Supplementary Table 1) electrofusion sample, amplified earlier (Cq 30) than the NTC but later than the coriander (Cq 24). Both samples consisted of an entire jar of plantlets pooled together in a mix (microplant samples, Fig. 3). All the other fusion derived samples, with ‘Dolanka’ or ‘Parmex’ carrot and coriander were negative for both *Atp1* and *Atp9*.

**Figure 4:**
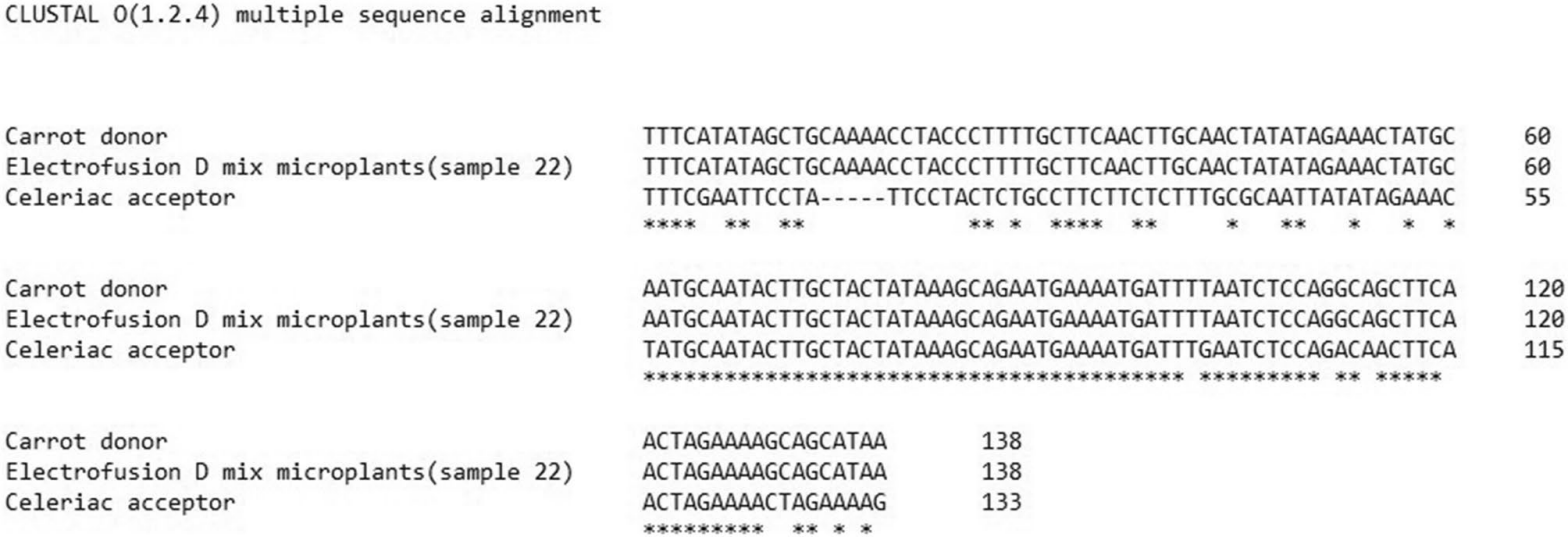
Alignment of the amplicon *Atp1* from the electrofusion D microplant sample to the ‘Diamant’ celeriac acceptor and ‘Dolanka’ carrot donor amplicons. The fusion derived samples is identical to the donor.

The 2 putative positive amplicons were sequenced. The sequence of the *Atp1* amplicon of the electrofusion D derived sample was identical to the *Atp1* amplicon of the donor ‘Dolanka’ carrot (Fig. 4) confirming the presence of some carrot mitotypes in the microplant sample. The *Atp9* amplicon of coriander fusion sample (sample 131) had a sequence similar to the acceptor ‘Diamant’ celeriac (Supplementary Fig. 1) indicating amplification profiles that did not correspond to donor mitotypes and were in fact amplified celeriac DNA. Amplification of *DcMP* gave a 1700 bp band in ‘Dolanka’ and ‘Parmex’ carrot, a 1700 bp and a 600 bp band in coriander and a 600 bp band in ‘Diamant’ celeriac and ‘WL253’ white celery. No distinction could be made between celeriac and white celery (Supplementary Fig. 2).

All fusion derived samples presented a single band of 600 bp similar to the celeriac acceptor band (Supplementary Fig. 3). In the plants resulting from carrot fusions, the 1700 bp band was present only in the positive control, namely the microplant pooled sample (no 2) resulting from electrofusion D (Supplementary Fig. 3). None of the plants resulting from fusions with coriander, contained the 1700 bp band, indicating a lack of coriander chloroplast integration in these plants (Supplementary Fig. 3).

## Discussion

The establishment of a protocol for asymmetric protoplast fusion of celeriac with related genera is an important step toward the creation of hybrids (fused nuclei) and cybrids (cytoplasmic hybrids). In the present work, we exposed the protoplasts to UV irradiation of 257 µW cm^-2^ for the donor nuclear genome inactivation. The UV effect on protoplast viability was consistent in the DNA fragmentation and fusion experiments, which confirms the high reproducibility of this technique, corroborating previous reports^16,17^.

Although we used genetically identical cell lines, the results of the IOA treatment varied between the biological repeats. The susceptibility to the IOA treatment varied from highly susceptible (cell line 23 and 25) to moderately susceptible (cell line 26) until highly tolerant (cell line 24). We observed variations in the effects of IOA treatment throughout the fusion experiments insofar the same cell line was used. We account this variation on the poor reproducibility of the IOA treatment caused by technical issues. We did not determine the exact cell number in the treated samples before IOA incubation because celery protoplast maintenance at high densities in salt-rich medium agglutinates all cells in a large clump. This results in incorrect cell density measurements and affects the level of cell exposure to IOA. Instead the optical density of the cell suspensions was used to prepare the same amount of cells in the enzymatic incubation phase throughout the experiments.

Terada, et al.^18^ used IOA to inactivate *B. oleracea* protoplasts and performed fusions with *B. campestris. B. oleracea* protoplasts were completely killed by 7.5 mM IOA but a much higher dose, 15 mM, was necessary to obtain 90% hybrid plants. They proposed that the tolerance of IOA-treated protoplasts in the fused sample is due to a ‘nursing effect’ exerted by the non-IOA treated donor cells. We applied a single IOA dose of 10 mM during different exposure times. For all cell lines, 20 min exposure time efficiently decreased cell viability. For experiment 5 we applied a more stringent exposure time, 25 min, to overcome possible restorative effects by co-cultivation with non-IOA treated donor cells.

Fusions with a lethal IOA treatment of acceptor protoplasts did not divide (with the exception of one electrofusion where the fused sample produced callus but no shoots), as there was no metabolic complementation of the acceptor by the donor. Similar results were obtained by Mollers and Wenzel^19^ who used a low IOA dose (3 mM for 15 min) to partially metabolically inactivate potato protoplasts, because they observed that doses that were lethal in the acceptor control arrested division initiation in the fused sample. We therefore performed the fusions using cell line 24, the most tolerant to IOA, to ensure the best chances towards shoot development. The lack of regeneration after successful celeriac:carrot or celeriac:coriander fusions could have multiple causes. First, incompatibilities between genome and cytoplasmome may result in hybrid breakdown of the fusion products, completely arresting cybrid growth. Second, in spite of the successful fusion, acceptor cells may not be sufficiently complemented with donor mitochondria and therefore remain metabolically inactive. For this reason, we executed a series of experiments, in which, besides the 2 putative CMS-inducing donor species, we used white celery as an additional donor. After intraspecific celeriac:white celery fusion, lack of regeneration can only be explained by lack of metabolic complementation and not by hybrid breakdown. In our experiments, the metabolic complementation did indeed not happen in any of the donor:acceptor combinations in the 3^rd^ electrofusion test (Experiment 4), after IOA treatment completely annihilated the celeriac protoplasts. This supports the hypothesis of insufficient metabolic complementation of the acceptor by donor mitochondria and we speculate that the higher the toxicity of the IOA treatment, the more transfer of donor mitochondria is required to enable cell division. Last, the fusion events could have been scarce and not enough cells lived long enough in the sample to support the division of the cybrids.

We tested a high number of fusion-derived plants for the presence of 3 mitochondrial markers. We used markers described in literature for carrot characterization, which explains why all the regions we tried to detect strongly amplified in carrot samples; the HRM *Cox1* was even able to distinguish the 2 carrot cultivars we used during distinct fusion experiments. *Atp1* and *Atp9* amplified in coriander as well. However, these carrot specific mitochondrial regions did not amplify in celeriac and white celery, indicating that the sequences are too divergent in these two species and thus could not be used for screening. A sample derived from carrot electrofusion D that consisted of a mixture of microplants gave an *Atp1* amplification with a high Cq but still below the NTC and the threshold of 35 cycles. Sequencing of this fragment confirmed the amplicon was the same as the *Atp1* of the donor carrot. It is possible that in the mixture of probably over 1000 plantlets, one or a few contained exclusively carrot cytoplasm, which were hence severely diluted in the sample that contained mainly celery cytoplasm. More plausibly, the cybrid plant(s) in the mixture contained both carrot and celeriac mitochondria or recombinant mitochondria, which is supported by the fact that the sample was negative for the other 2 markers tested.

The amplification of the *DcMP* in coriander resulted in formation of a strong 1700 bp band and a weaker 600 bp band in contrast with the 400 bp amplicon reported by Iorrizo et al. 2012^20^. Through *DcMP* amplification, we could not detect the presence of donor chloroplasts in any other fused sample. The data on chloroplast screening confirm our data on mitochondrial screening, as carrot *Atp1* was found in the fusion D sample that had carrot *DcMP*.

Our study demonstrates the utility of mitochondrial markers for characterization of somatic fusion between celery and other Apiaceae. The presence of both mitochondrial and chloroplast markers in a fused sample points out that there is a simultaneous transfer of as well mitochondrial as chloroplast DNA during protoplast fusion. The occurrence of a regenerated fusion product also corroborates the successful induction of hybrid cells. The low frequency of hybrid shoot formation suggests a mitochondrial segregation in favor of the original or the most prevalent type or lack of donor mitochondria integration. Immediately after the fusion, donor fragments may have been integrated in celeriac mitochondria, or intact donor mitochondria could have been inserted. If the number of donor or hybrid mitotypes is lower than the celeriac mitotype, there is a great chance that the lower fraction would segregate out of the mitochondrial population. Segregation of mitotypes in favour of one of the partners was also observed in symmetric somatic hybrids between chicory and endive^17^. Since only 3 mitochondrial regions were checked, we may have missed other mitochondrial regions integrated in the acceptor background. Future experiments should include sequencing of the complete mitochondrial genome, and this should allow creating more specific markers for discriminating the parent genomes.

## Material and methods

### Plant material

A celeriac ‘Diamant’ and a white celery ‘Daybreak’ plant regenerated from protoplasts were used for the induction of a single callus line from leaf material as described earlier^11^. One ‘Daybreak’ and 4 ‘Diamant’ independent cell suspension lines (23, 24, 25 and 26) were generated by inoculating 3 g of friable callus in 30 ml liquid Murashige and Skoog medium (MS). Cultures were maintained weekly through 1:1 dilution in an 250 ml Erlenmeyer flask and incubated in the dark at 22 ± 2 °C with continuous agitation (100 rpm).

Seedlings of the white celery inbred line ‘WL253’, the open pollinated carrot varieties ‘Parmex’, ‘Dolanka’ and ‘Karotan’ and the open pollinated coriander with accession code csR1 (ILVO collection) were cultivated in vitro. Seeds were pre-soaked for 10 min in water at 50 °C and surface sterilized in 70% ethanol for 1 (coriander) or 5 min (celery and carrot), followed by 15 min in 0.75% NaOCl with 2 drops l^-1^ Tween 20 (Duchefa Farma BV). Seeds were then rinsed 3 times in sterile distillate water and sown in a Meli jar containing solid MS with vitamins supplemented with 20 g l^-1^ sucrose and 6 g l^-1^ plant tissue culture agar (Duchefa Farma BV). The pH of the culture media was adjusted to 5.8 prior to autoclaving unless stated otherwise. The Meli jars were incubated at 22 ± 2 °C, with 16 h photoperiod (PPFD 40 μmol m^-2^ s^-1^), for seed germination and plantlet growth.

### Protoplast isolation

Celeriac protoplasts were isolated from 7 to 11-week-old cell suspension cultures as described by Bruznican, et al.^11^. The protoplasts were washed with either 10 ml modified W5 (for IOA treatment testing) or 10 ml 0.5 M mannitol solution (for fusion) and centrifuged for 10 min at 100 g, 8 °C.

Carrot, coriander and white celery protoplasts were isolated from petioles and leaves of 4 to 6-week-old plantlets. Fresh tissue (500 mg) was harvested in 5 ml EKW buffer (0.1% CaCl_2_·2H_2_O, 0.06% MES and 13% sucrose), to prevent tissue dehydration^21^. When the harvesting was completed, the EKW buffer was replaced with 10 ml of enzyme solution, consisting of 1.5% cellulase R10 (Duchefa Farma BV), 0.1% macerozyme (Duchefa Farma BV) and 300 mg l^-1^ cefotaxime (Duchefa Farma BV) dissolved in EKW buffer. The samples were cut in small pieces before overnight incubation at 22 °C while gently shaking (30 rpm) in the dark.

After digestion, the samples were filtered through 70 μm (Falcon) and 40 μm (SPL Life Sciences) nylon sieves, washed with 30 ml W5^22^ and centrifuged for 10 min at 100 g, 8 °C. The protoplasts contained in the pellet were then washed in 10 ml 0.6 M sucrose solution overlaid with 1 ml W5 medium and centrifuged for 10 min at 80 g, 8 °C. The viable protoplasts floating in a band between the two media were collected, washed with 10 ml W5 and centrifuged for 5 min at 100 g, 8 °C. The protoplasts were washed with either 10 ml modified carrot petiole protoplast (CPP) culture medium [macro-, micro-elements and organic acids according to Kao and Michayluk^23^, vitamins according to Gamborg, et al.^24^, 74 g l^-1^ glucose, 250 mg l^-1^ enzymatic casein hydrolysate, 0.3 mg l^-1^ 2,4-D and 0.012 mg l^-1^ TDZ, pH 5.6] when used for testing UV treatments, or with 10 ml 0.5 M mannitol when used for fusions, and centrifuged for 10 min at 100 g, 8 °C.

For all protoplast types, the total number of isolated cells was determined using a Bürker haemocytometer chamber, and the density was adjusted to 10^6^ protoplasts per ml with 0.5 M mannitol or CPP medium.

### Protoplast fragmentation and fusion

#### Experiment 1: UV inactivation of donor protoplasts

‘Parmex’ carrot, coriander and ‘WL253’ white celery protoplasts, suspended in modified CPP medium, were distributed in 55 mm plastic Petri dishes in 2 ml volumes. The protoplasts were exposed to the UV light (Sylvania G30T8/OF, UVC output of 13.4 W at 254 nm) for 0, 2, 4 and 6 min in opened dishes. UV-irradiated protoplasts were carefully protected from the light during all following manipulation steps. Following irradiation, the protoplasts were cultured at a density of 2 × 10^5^ protoplasts per ml in modified CPP medium.

#### Experiment 2: IOA inactivation of acceptor protoplasts

The purified celeriac protoplasts of ‘Diamant’ cell lines 23, 24, 25 and 26 were exposed to 10 mM IOA dissolved in modified W5 solution, in 20 ml volumes, in the dark, on ice for 0, 10, 15 and 20 min. After the treatment, the protoplasts were washed once with 30 ml and two times with 50 ml modified W5 and centrifuged 4 min at 100 g, 8 °C. The washed protoplasts were rinsed in 50 ml modified CPP medium and centrifuged 15 min at 100 g, 8 °C. The IOA-treated protoplasts were cultured at a density of 2 × 10^5^ protoplasts per ml in modified CPP medium.

#### Microscopic evaluation of experiment 1 and 2

IOA and UV effects were examined using a Leica DMi8 inverted microscope equipped with a Leica DFC450C camera and an UV lamp. For both donor and acceptor protoplasts, cell viability was assessed by staining 100 µl culture with 1 µl 0.5% FDA (5 mg FDA dissolved in 1 ml acetone). Pictures of the FDA stained cells were taken in both bright field and under UV light with the appropriate filter, immediately after treatment and 7 days later. The treatments were independently repeated in 4 biological repeats that were set up in 4 technical replicates. Per technical replicate approximately 300 cells were counted in bright field (BF) and the viability frequency was calculated (no of FDA stained cells *100/no of cells observed in BF). The mean values and standard errors of the viability frequency were calculated. The assumption of the homogeneity of the variance for a two factorial ANOVA was not fulfilled. Hence a one-way ANOVA within the species/cell line was conducted and in case all ANOVA’s assumption were fulfilled, a Tukey’s honestly significant difference (HSD) test was performed. When the homogeneity of the variance was violated, a Welch’s robust tests of equality of means was employed. All statistical analyses were conducted using SPSS Statistics version 25.

#### Experiment 3: Fluorescent markers to monitor fusion events

To confirm fusion, 1 ml ‘Diamant’ celeriac cell line 24 acceptor protoplasts was stained with 1.25 µl 0.5% FDA (5 mg FDA dissolved in 1 ml acetone) and 1 ml donor protoplasts (‘WL253’ celery, ‘Parmex’ carrot, coriander) was stained with 20 µl of 2 mM Calcein Blue AM (Invitrogen) dissolved in DMSO. The density of the stained samples was 10^6^ protoplasts per ml. After 10 min incubation with the dyes, the donor and acceptor cells were mixed 1:1 and were chemically or electrically fused as described in Experiment 5. The stained fused and unfused cells were visualized using a fluorescence microscope Axio Imager 2 (Carl Zeiss AG) equipped with a filterset 10 (excitation 450–490, emission 515–565) and filterset 02 (excitation 365, emission 420) (Carl Zeiss AG). The pictures were taken at 200 × magnification using an AxioCam MRm camera and the Zen software (Carl Zeiss AG).

#### Experiment 4: preliminary protoplast fusions

To evaluate the effect of IOA and UV treatment on regeneration, a first series of preliminary asymmetric electrofusion experiments were performed as described in Experiment 5, using various donor genera and genotypes and celeriac and white celery acceptor cell lines. The duration of UV and IOA treatments, the number of fusion pulses and the ratio between donor and acceptor protoplasts varied between fusions. All fusion parameters are indicated in Table 2.

#### Experiment 5: subsequent protoplast fusions

All fusion experiments were performed with the ‘Diamant’ celeriac cell line 24 as acceptor. The age of the cell line 24 was between 28 and 33 weeks old for the electrofusion experiments and between 41 and 46 weeks old for the PEG fusion experiments. The acceptor protoplasts treated with 10 mM IOA for 25 min were suspended in 0.5 M mannitol at a density of 10^6^ protoplasts per ml. Donor protoplasts of ‘WL253’ white celery, ‘Parmex’ carrot and coriander at 10^6^ protoplasts per ml in 0.5 M mannitol were UV-irradiated for 6 min. Three 1:1 mixtures of celeriac:carrot, celeriac:coriander and celeriac:white celery were made. Besides the fusion treatment (electrical or chemical) each time also non fused and treated or non-treated acceptor and donor protoplasts were cultured as controls. All the fused cells and controls were cultured at a density of 2 × 10^5^ protoplasts per ml in modified CPP medium supplemented with 25% condition medium, as described by Bruznican, et al.^11^ to a final 1 ml volume in 35 mm Petri dishes and incubated in the dark at 22 ± 2 °C. Both the electrical and chemical fusion experiments were repeated 4 times independently.

Electrofusion was performed using an Eppendorf Multiporator equipped with an Eppendorf helix fusion chamber, with a gap of 200 µm between the platinum electrodes. 250 µL of the protoplast mixture was pipetted in the cuvette and an alternating current (AC) of 3.5 V was applied for 40 s during both alignment and post-alignment steps. In between, 2 direct current (DC) pulses of 25 V were applied for 60 µs to induce cell fusion. For each protoplast mixture, 8–10 fusions were performed, and the recovered cells were washed together in 10 ml CPP medium and centrifuged for 15 min at 100 g, 8 °C.

Chemical fusion was performed using a fusion solution [400 g l^-1^ polyethylene glycol (PEG, MW 3350), 72.8 g l^-1^ mannitol and 23.6 g l^-1^ Ca(NO_3_)_2_·4H_2_O dissolved in water and filter sterilized (0.2 μm, Whatman)].The protoplast mixtures were distributed in 150 µl drops among 10 Petri dishes (5.5 mm diameter). Two drops of 75 µl PEG solution were placed next to each protoplast drop. The fusion was initiated by merging the 3 drops, followed by a 30 s incubation time. Subsequently, 3 ml of 0.5 M mannitol was added to the fusion mixture in each Petri dish and the whole was incubated for 10 min. The contents of 2 Petri dishes were mixed and washed in 6 ml modified W5 solution, followed by centrifugation for 5 min, at 100 g, 8 °C. The resulting pellets were placed into two 12 ml tubes and washed in 10 ml modified CPP, followed by a centrifugation step for 15 min, at 100 g, 8 °C.

Based on the number of cells used for fusion and the number of cells recovered after fusion and washing steps, the post-fusion cell recovery percentage was calculated. The recovery percentage of as well different fusion types (chemical fusion vs electrofusion) and partner combinations (carrot, coriander or white celery as donor partner) were determined.

### Somatic hybrid regeneration

The osmolarity of the protoplast culture medium was decreased stepwise as described by Bruznican, et al.^11^. Five weeks after chemical fusion and 6 weeks after electrofusion, the cultures were transferred on a nylon mesh with 50 µm apertures layered on solid CPPD medium modified from Dirks, et al.^21^. The cultures were maintained in the dark and refreshed every 2 weeks on fresh modified CPPD medium, until the first shoots started to appear. Starting with 2 months after protoplasts fusion, the forming shoots were placed in glass jars on the seed germination medium and exposed to a 16 h photoperiod (PPFD 40 μmol m^-2^ s^-1^) regime at 22 ± 2 °C.

### Molecular characterization

Molecular characterization of the putative somatic hybrids was performed 5–9 months after the fusion. To reduce the workload, we pooled plantlets by 5 plantlets per jar. When the plantlets were too small to provide enough material, the entire content of a Weck jar was pooled in a sample, further referred to as microplant sample, which could not be further analysed individually (Fig. 3).

DNA was isolated from 100 mg of fresh tissue using the cetyltrimethylammonium bromide (CTAB) method according to Doyle and Doyle^25^ with the following modifications: 0.4% β-mercaptoethanol were used in the CTAB buffer instead of 0.2%, 1 volume of isopropanol was added to dissolve DNA instead of 2/3 and a second washing step in 76% EtOH/10 mM ammonium acetate was performed. DNA concentration was determined using a NanoDrop ND-1000 spectrophotometer (Isogen Life Science) and was diluted with mQ water to a final concentration of 15 ng µL^-1^ prior to polymerase chain reaction (PCR) amplification.

HRM analysis was used to detect different PCR amplicons of the mitochondrial genes *Cox1*, *Atp1* and *Atp9* between celeriac ‘Diamant’ cell line 24 acceptor plants and coriander, carrot (‘Parmex’ and ‘Dolanka’) and ‘WL253’ white celery donor plants. Likewise, amplicon length differences in the chloroplast gene *DcMP* were compared. For the PCR amplification, the primers depicted in Table 6 were used.

**Table 6:** Primer set used for molecular characterization of somatic hybrids.

Gene	Forward	Reverse	References
*Cox1*	AGTGATGGGCACATGCTTTT	TCATCGCAGGCATAACCATA	(Mandel, unpublished)
*Atp1*	GCAAGAAGGAAAGCTGTTAGAG	GGTATCCCTCTTCTGTTTCGG	^26^
*Atp9*	GAAGGTGCAAAATCAATAGG	TACATGGACTTTAAATTGACTTCT	^26^
*DcMP*	AAAGGAATTTGTCCATTTTTC	GCCTCTTCTTGATTCTCGTT	^20^

The HRM reaction was carried out in a 384 well plate in a final 10 µl reaction mix containing 2 × SensiFast HRM Master Mix (Bioline), 0.2 µM forward and reverse primers and 30 ng DNA. The PCRs were performed in a LightCycler 480 Real-Time PCR (Roche Diagnostics) starting with a denaturation at 95 °C for 3 min, followed by 45 cycles of 95 °C for 5 s, 60 °C for 10 s and 72 °C for 20 s. Melting profiles were determined as follows: 1 min 95 °C, 1 min 40 °C, 65 °C for 1 s and a melting step from 65 to 95 °C with a ramp rate of 0.02 °C s^-1^. The fluorescence signal data were recorded continuously, and the melting profile of individual curves were analysed and grouped using the LightCycler 480 Gene Scanning Software (Roche Diagnostics). All samples were tested in duplicate and a no template control (NTC) was included. In case the PCR amplification of specific genes could differentiate between donor and acceptor plants, putative somatic hybrid samples were analysed with these markers as well (Table 5).

For *DcMP*, the validation of the donor and acceptor plants was already described^27^. The PCR reaction was carried on as described in that paper. Two subsets of regenerants from experiment 4 (electrofusion D and G), were reported in our previous publication^27^ and are in this paper included as positive (electrofusion D) and negative (electrofusion G) control samples.

In case donor amplicons were detected in the putative somatic hybrids, the PCR fragments were purified by adding 1U *exo* I (NEB) and 2U FastAP (Thermo Fisher Scientific) to 10 µl of PCR product. Samples were incubated in a PCR machine for 15 min at 37 °C and 15 min at 85 °C followed by cooling at 4 °C. Purified DNA (50–250 ng) was Sanger sequenced using 25 pmol gene specific primer by GENEWIZ Germany GmbH. Clustal Ω^28^ was used to confirm fragment identity.

## Supplementary Information

Supplementary Information

Supplementary Legends.
