# Supplementary material for: An asymmetric protoplast fusion and screening method for generating celeriac cybrids
Source: Sci Rep. 2021 Feb 25;11:4553. doi: 10.1038/s41598-021-83970-y (PMC7907277; doi:10.1038/s41598-021-83970-y)
Supplement: Supplementary file 2 — Supplementary Legends. [file 41598_2021_83970_MOESM2_ESM.docx]

**Supplementary figure 1.** Alignment of the amplicon *Atp9* from electrofusion 2 with coriander derived samples (Experiment 5) to the ‘Diamant’ celeriac acceptor and coriander donor amplicons. The fusion derived samples are identical to the acceptor.

**Supplementary figure 2.** Amplification of *DcMP* region in donor and acceptor plants used during the fusions. Lanes **1- 6** are coriander donor plants, lanes **7- 11** are carrot ‘Parmex’ donor plants, lanes **12- 15** are ‘WL253’ white celery donor plants, lane **16** is a ‘Diamant’ celeriac acceptor plant, lane **17** is a carrot ‘Dolanka’ donor plant and lane **18** is the negative control. M is the marker ladder (100bp Plus).

**Supplementary figure 3.** Amplification of *DcMP* region in samples derived from fusions. The lanes represent: **1** is a sample from fusion F (negative control of fusion with carrot ‘Parmex’); **2** is a sample from fusion D (positive control from fusion with carrot ‘Dolanka’); **3** is a carrot ‘Parmex’ donor plant; **4** is a carrot ‘Dolanka’ donor plant; lanes **5- 8** are samples of microplants from electrofusions with coriander; lanes **9 -16** are samples of microplants from PEG fusions with coriander; **17- 21** are coriander donor plants and lane **22** is a ‘Diamant’ celeriac acceptor plant. M is the marker ladder (100bp Plus).

**Supplementary table 1.** The Cq values of *Atp1* and *Atp9* amplicons detected in fusion regenerants and controls.
